# Exercise Combined with *Rhodiola sacra* Supplementation Improves Exercise Capacity and Ameliorates Exhaustive Exercise-Induced Muscle Damage through Enhancement of Mitochondrial Quality Control

**DOI:** 10.1155/2017/8024857

**Published:** 2017-11-22

**Authors:** Yaoshan Dun, Suixin Liu, Wenliang Zhang, Murong Xie, Ling Qiu

**Affiliations:** Cardiac Rehabilitation Center, Department of Rehabilitation, Xiangya Hospital of Central South University, Changsha 410008, China

## Abstract

Mounting evidence has firmly established that increased exercise capacity (EC) is associated with considerable improvements in the survival of patients with cardiovascular disease (CVD) and that antistress capacity is a prognostic predictor of adverse cardiovascular events in patients with CVD. Previous studies have indicated that aerobic exercise (AE) and supplementation with *Rhodiola sacra* (RS), a natural plant pharmaceutical, improve EC and enable resistance to stress; however, the underlying mechanism remains unclear. This study explored the ability of AE and RS, alone or combined, to improve EC and ameliorate exhaustive exercise- (EE-) induced stress and elucidate the mechanism involved. We found that AE and RS significantly increased EC in mice and ameliorated EE-induced stress damage in skeletal and cardiac muscles (SCM); furthermore, a synergistic effect was detected for the first time. To our knowledge, the present work is the first to report that AE and RS activate mitophagy, mitochondrial dynamics, and biogenesis in SCM, both in the resting state and after EE. These data indicate that AE and RS synergistically improve EC in mice and protect SCM from EE-induced stress by enhancing mitochondrial quality control, including the activation of mitophagy, mitochondrial dynamics, and biogenesis, both at rest and after EE.

## 1. Introduction

Cardiovascular disease (CVD) is the leading cause of disease death worldwide [1]. It has been firmly established that low level of exercise capacity (EC) is associated with cardiovascular disease mortality and all-cause mortality in patients with CVD [2]. A growing body of epidemiological and clinical evidence demonstrates that EC is a potentially stronger predictor of mortality than established risk factors such as smoking, hypertension, high cholesterol, and type 2 diabetes mellitus [3, 4]. Moreover, numerous recent studies have shown that each 1 MET increment (MET, a multiple of the resting metabolic rate approximating 3.5 ml·kg^−1^·min^−1^) in EC is associated with considerable (10%–25%) improvement in survival [5]. A recent scientific statement from the American Heart Association recommended the use of EC as a clinical vital sign [5]. In addition, antistress capacity is currently used as prognostic predictors of major adverse cardiovascular events, including cardiac and all-cause death, nonfatal myocardial infarction, and coronary revascularization, PTCA/CABG, in patients with CVD [6–8]. Kaplan-Meier survival estimates showed a significantly worse outcome in patients presenting with elevated oxidative stress levels [8]. Improved antistress capacity was found to reduce the area of skeletal muscle damage after ischemia or hypoxia [9] as well as the incidence of malignant ventricular arrhythmia after a previous myocardial infarction [10]. Therefore, the development of strategies to improve EC and the capacity to resist acute stress-induced damage are of great clinical significance. This study examined the ability of both nondrug intervention-based and pharmaceutical supplementation to enhance EC and the capacity to resist acute stress-induced damage, with a focus on aerobic exercise (AE) and supplementation with *Rhodiola sacra* (RS), a traditional natural plant pharmaceutical.

EC reflects the integrated ability to transport oxygen from the atmosphere to the mitochondria to perform physical work. It therefore quantifies the mitochondrial function of an individual and is dependent on a linked chain of processes that include pulmonary ventilation and diffusion [11], right and left ventricular functions [12], and the ability of skeletal and cardiac muscle (SCM) cells to receive and use the oxygen and nutrients delivered by the blood [13]. In addition, mitochondria are multifunctional organelles whose quality is closely related to antistress capacity [14]. Thus, the mitochondrial quality in SCM is the prime factor influencing EC and the degree of acute stress-induced damage in an individual. However, few studies have been performed on the relationship between the mitochondrial quality in SCM and EC and the ability to resist acute stress-induced muscle damage. Mitochondrial quality control (MQC) functions on molecular, organellar, and largely intraorganellar levels. On the organellar level, there is an interplay among mitophagy, mitochondrial dynamics, and biogenesis [15].

On the one hand, mitochondrial fission, a component of mitochondrial dynamics, combined with mitophagy promotes the isolation and elimination of damaged mitochondrial components [15]; this process is vital for the maintenance of cell homeostasis. As oxidative stress increases and damaged mitochondria accumulate, fission, mediated by dynamin-related protein-1 (DRP1) [16], isolates damaged components for elimination. Mitochondrial depolarization induced by damage allows for the transposition of BCL2/adenovirus E1B 19 kDa protein-interacting protein 3 (BNIP3) to the mitochondrial membrane as a target of the autophagosome [17]. p62 also plays a role in targeting cargo to the autophagosome and is subsequently degraded during autophagy and mitophagy [18]. Assembly of the phagosome involves the conjugation of microtubule-associated protein 1 light chain 3 (LC3) with phosphatidylethanolamine to form LC3-II. On the other hand, mitochondrial fusion, another component of mitochondrial dynamics, combined with mitochondrial biogenesis produces new mitochondria. Mitochondria biogenesis is regulated by the AMP-activated protein kinase (AMPK)/peroxisome proliferator-activated receptor-*γ* coactivator 1*α* (PGC-1*α*) signaling pathway [19] through its activation of nuclear respiratory factor (NRF)-1, NRF-2, transcription factor A mitochondrial (TFAM), and transcription factor B mitochondrial (TFBM) [20].

AE is widely recognized as an effective approach to increase EC. However, further studies are required to determine the underlying mechanism. Several recent studies have suggested that AE ameliorates age-associated deterioration in mitochondrial biogenesis in rats [21], activates skeletal muscle autophagy [22], and triggers the AMPK/PGC-1*α* signaling pathway [23], which is pivotal for the regulation of mitochondrial biogenesis [24]. In addition, numerous lines of evidence indicate that regular exercise provides cardioprotection [25] and reduces chemical substance-induced oxidative stress and proteolysis in skeletal muscle [26]. However, few studies have been performed on the effect of AE on protecting SCM with a focus on MQC.

The combination of nondrug intervention (exercise)-based and pharmaceutical supplementation is commonly used in clinical practice. Natural plant pharmaceuticals have fewer side effects and higher acceptability than synthesized chemical drugs. RS, a traditional natural plant pharmaceutical, is widely distributed at high altitudes in the Arctic and mountainous regions throughout Europe and Asia [27]. For the last century, this plant has been well known for its physiological benefits such as fatigue elimination and prevention of high-altitude sickness [28]. Mounting evidence demonstrates that RS enhances exercise performance [28] and prolongs lifespan in *Drosophila melanogaster* [29] and silkworms [30] and ameliorates the oxidative stress induced by EE in rats [31]. Two recent studies have demonstrated that RS activates autophagy in bladder cancer cells [32]; further, this plant has been shown to promote mitochondrial biogenesis and reduce apoptosis induced by hydrogen peroxide in endothelial cells [33]. However, only a few studies have been performed to investigate the effects of RS supplementation on EE-induced muscle damage; further, none of these studies has addressed the effects of RS supplementation on enhancing MQC in SCM.

In this study, we explored the effects of AE and RS supplementation, alone and combined, on EC in mice and on protection against EE-induced SCM stress and then investigated their respective mechanisms with a focus on MQC, including mitophagy, mitochondrial dynamics, and biogenesis.

## 2. Materials and Methods

### 2.1. Ethics Statement

All animal protocols were approved by the Hunan Provincial People's Hospital Animal Care and Use Committee under the guidelines of the Chinese Academy of Sciences (approval ID: SYXK 2015-0013).

### 2.2. *Rhodiola sacra* (RS)

Highly pure extract from the root of RS was provided by Tibet Rhodiola Pharmaceutical Holding Company. HPLC-MS analysis revealed that the main effective contents are salidroside (C_14_H_20_O_7_, 2.62%) and flavone (C_27_H_30_O_16_, 3.27%). The preparation and inspection of the extract complied with The Chinese Pharmacopoeia 2015 (inspection report number C1051612067). To prepare a solution of the extract, extract powder was mixed with distilled water (50 mg/ml). The dosage was based on the weight of each mouse, at a liquid/weight ratio of 0.1 ml/10 g. The mice were administered with the extract by gavage every morning between 9 a.m. and 10 a.m. for five consecutive weeks.

### 2.3. Animal and Study Design

Male C57BL/6J mice (8 weeks old) were purchased from Hunan SJA Laboratory Animal Co. Ltd. (Changsha, Hunan, China), certification number SCXK 2011-0003. The mice were housed in temperature-controlled (22 ± 2°C) quarters with a 12 : 12 h light-dark cycle with free access to water and food. The mice were allowed to adapt to the conditions and fed for 1 week before the experiment. The mice were then divided into the control (Con, *n* = 10), placebo (P, *n* = 10), aerobic exercise (AE, *n* = 10), *Rhodiola sacra* (RS, *n* = 10), and exercise combined with *Rhodiola sacra* (AE + RS, *n* = 10) groups. The mice in the P group were administered with normal saline at a dose of 0.1 ml/10 g weight, those in the RS group were given *Rhodiola sacra* solution as described previously, and those in the exercise group performed an AE training program (see below for details). After the 5-week experimental period, each group was randomly and equally divided into normal (*n* = 5) and exhaustive exercise (EE, *n* = 5) subgroups, and the mice in the EE subgroups completed an EE protocol (see below for details) prior to sacrificing, after about 12 h. All mice were anesthetized via an intraperitoneal injection of 5% chloral hydrate (0.1 ml/10 g weight) and then sacrificed after taking a blood sample by removing the eyeball.

### 2.4. Animal Aerobic Exercise Training Protocol

Mice in the AE and AE + RS groups underwent a moderate intensity swim training protocol, as described previously [31], with modifications. The mice were placed in a Morris water maze pool (type number XR-XM101-R, 60 cm high, 120 cm in diameter) with a water depth of 30 cm maintained at 30 ± 2°C. The mice initially swam freely for 10 min on the first day; swimming time was then gradually increased to 60 min/day by adding 10 min each day. Then, the swim training consisted of 4 weekly sessions of 60 min of forced swimming with the mice tethered to a stick with string, 5 d/week. After the sessions, the mice were dried gently with towels and a blower and returned to their cages. To avoid circadian variations in physical activity, the swimming exercise sessions were performed between 9 a.m. and 2 p.m., when the mice have been previously confirmed to exhibit minimal variations in aerobic capacity [34].

### 2.5. Exhaustive Exercise (EE) Protocol

A previously described EE protocol was used with some modifications [35]. The mice in the EE subgroups (*n* = 25, 5 per group) performed a forced weight-loaded swimming session. The load (5% of their body weight) was composed of a lead sheath (0.8 mm thick, 0.5 cm wide) attached to the tail root of the mouse, which was then gently placed in the water. The conditions and equipment used in the test were consistent with those used in the AE training protocol. The mice were then made to swim until exhaustion, as defined as the failure to rise to the surface of the water to breathe within a 7-second period. All mice underwent a preadaptation period 2 days before the formal protocol.

### 2.6. Assessment of Exercise Capacity

The mice in the EE subgroups (*n* = 25, 5 per group) performed the EE protocol, and the duration of forced weight-loaded swimming until exhaustion was recorded as a measure of EC.

### 2.7. Measurement of the Function of Mitochondria

To determine whether AE and RS, alone and combined, enhance the function of mitochondria in mouse SCM, we analyzed the expression of citrate synthase (CS). CS, the first and rate-limiting enzyme of the tricarboxylic acid cycle, plays a key role in regulating energy production during mitochondrial respiration [36].

### 2.8. Measurement of Exhaustive Exercise-Induced Skeletal and Cardiac Muscle Damage

To determine the degree of muscle damage, serum samples from mice in the EE subgroup were assayed for creatine kinase (CK) using an assay kit (A032, Nanjing Jiancheng Bioengineering Institute, China), as CK is a marker of damage in CK-rich tissues such as SCM [37]. In addition, to further confirm the evidence of EE-induced muscle damage and assess the degree of muscle damage, the morphology of muscle fibers and mitochondria was observed using transmission electron microscopy (Tecnai G2 Spirit, FEI, USA).

### 2.9. Assessment of the Level of Mitochondrial Oxidative Stress in Skeletal and Cardiac Muscles

The xanthine oxidase method was used to determine the activity of manganese superoxide dismutase (MnSOD) in skeletal (gastrocnemius) and cardiac (left ventricle) muscles, according to the manufacturer's instructions (A001-2, Nanjing Jiancheng Bioengineering Institute, China). The thiobarbituric acid (TBA) method was applied to detect the mitochondrial content of malondialdehyde (MDA) in skeletal (gastrocnemius) and cardiac (left ventricle) muscles, according to the manufacturer's instructions (A003-2, Nanjing Jiancheng Bioengineering Institute, China).

### 2.10. Isolation of Mitochondria from Skeletal and Cardiac Muscles

The mitochondria from skeletal (quadriceps femoris) and cardiac (left ventricle) muscles of the mice were isolated using differential centrifugation according to established protocols (G006, Nanjing Jiancheng Bioengineering Institute, China).

### 2.11. Assessment of Autophagy and Mitophagy

Autophagy and mitophagy in skeletal (quadriceps femoris) and cardiac (left ventricle) muscles were analyzed by Western blotting of tissue extracts with antibodies against LC3 (microtubule-associated protein 1 light chain 3), p62 (SQSTM1, sequestosome), and BNIP3 (BCL2/adenovirus E1B 19 kDa protein-interacting protein 3). TEM was used to further confirm evidence of autophagy.

### 2.12. Assessment of Mitochondrial Dynamics

Mitochondrial dynamics in skeletal (quadriceps femoris) and cardiac (left ventricle) muscles was analyzed by Western blotting of tissue extracts with antibodies against DRP1 and MFN1.

### 2.13. Assessment of Mitochondrial Biogenesis

The activation of the AMPK/PGC-1*α* signaling pathway in skeletal (quadriceps femoris) and cardiac (left ventricle) muscles was analyzed by Western blotting and RT-qPCR.

### 2.14. Protein Assay

For protein content analysis, skeletal (quadriceps femoris) and cardiac (left ventricle) muscle samples were homogenized and analyzed by Western blotting. The following antibodies were used: BNIP3 (ab109362, rabbit, 1 : 1000), p-AMPK (ab131357, rabbit, 1 : 500) from Abcam (USA); PGC-1*α* (sc-13067, rabbit, 1 : 200) from Santa Cruz (USA); CS (16131-1-AP, rabbit, 1 : 1000), LC 3 I/II (14600-1-AP, rabbit, 1 : 500), AMPK (10929-2-AP, rabbit, 1 : 500), DRP1 (12957-1-AP, rabbit, 1 : 500), MFN1 (13798-1-AP, rabbit, 1 : 500), and p62 (18420-1-AP, rabbit, 1 : 1000) from Proteintech (USA); and GAPDH (AP0063, rabbit, 1 : 5000) from Bioworld (USA). Membranes were analyzed and quantified using the Quantity One Imaging System (BIO-RAS, USA). Protein expression was normalized to that of GAPDH.

### 2.15. Gene Expression

Total RNA was extracted from skeletal (quadriceps femoris) and cardiac (left ventricle) muscles (TRIzol, Invitrogen, CA, USA). AMPK and PGC-1*α* mRNA levels were quantified by real-time reverse transcription PCR (RT-qPCR) analysis and normalized to those of GAPDH. Primer sequences are listed in Table 1.

### 2.16. Statistical Analyses

Data are presented as mean ± SD. A single-factor analysis of variance (ANOVA) was used to analyze the results of the EE test, as well as serum CK, protein, and gene expression levels. Statistical analyses were conducted using SPSS statistical software 21.0 (IBM, USA). A significance level of 0.05 was used for all analyses.

## 3. Results

### 3.1. Effects of Aerobic Exercise and *Rhodiola sacra*, Alone and Combined, on Mice Exercise Capacity and the Underlying Mechanism

#### 3.1.1. AE and RS, Alone and Combined, Increase Mice Exercise Capacity

**Figure 1:**
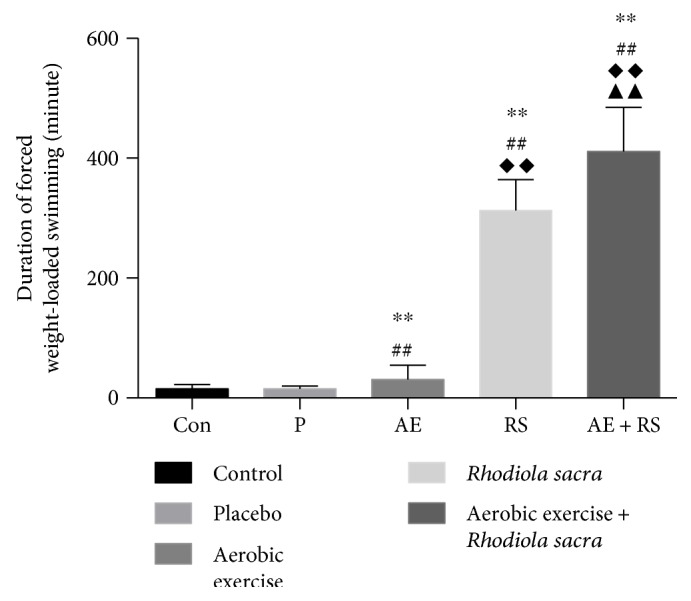
Effects of aerobic exercise and *Rhodiola sacra* supplementation, alone and combined, on the duration of forced weight-loaded swimming in mice; Con, control; P, placebo; AE, aerobic exercise; RS, *Rhodiola sacra*; AE + RS, aerobic exercise combined with *Rhodiola sacra*; ^∗∗^*P* < 0.01 versus Con; ^##^*P* < 0.01 versus P; ^◆◆^*P* < 0.01 versus AE; ^▲▲^*P* < 0.01 versus RS. *n* = 5 mice. All data are expressed as mean ± SD.

The duration of forced weight-loaded swimming until exhaustion was recorded as a measure of EC, as described previously. A significantly increased duration of swimming was observed in the AE group compared with that in the Con group (30.60 ± 24.22 versus 15.25 ± 7.18 min, *P* < 0.01, *n* = 5). Similarly, the duration of swimming was twenty times longer following in mice administered RS compared with those that received the placebo (312.60 ± 51.61 versus 15.00 ± 4.97 min, *P* < 0.01). The combination of AE and RS further significantly increased the duration of swimming compared with AE or RS alone (*n* = 5 mice, *P* < 0.01) (Figure 1).

#### 3.1.2. AE and RS, Alone and Combined, Enhance the Function of Mitochondria in Skeletal and Cardiac Muscles

**Figure 2:**
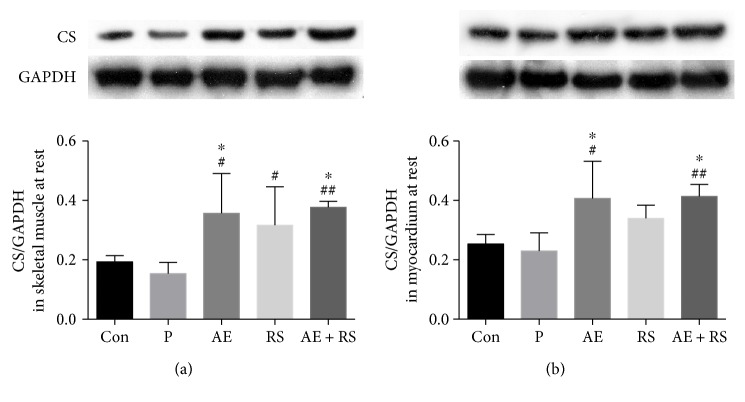
Effects of aerobic exercise and *Rhodiola sacra* supplementation, alone and combined, on the expression of citrate synthase (CS) in mouse skeletal (a) and cardiac muscles (b). ^∗^*P* < 0.05 versus Con; ^#^*P* < 0.05, ^##^*P* < 0.01 versus P.

To determine whether AE and RS, alone and combined, enhance the function of mitochondria in mouse SCM, we analyzed the expression of CS. Both AE and RS supplementation significantly increased the level of CS in SCM (*P* < 0.05). The combination of AE and RS supplementation further increased the expression of CS in SCM (*P* < 0.05) (Figure 2).

#### 3.1.3. AE and RS, Alone and Combined, Activate Autophagy and Mitophagy in Skeletal and Cardiac Muscles

**Figure 3:**
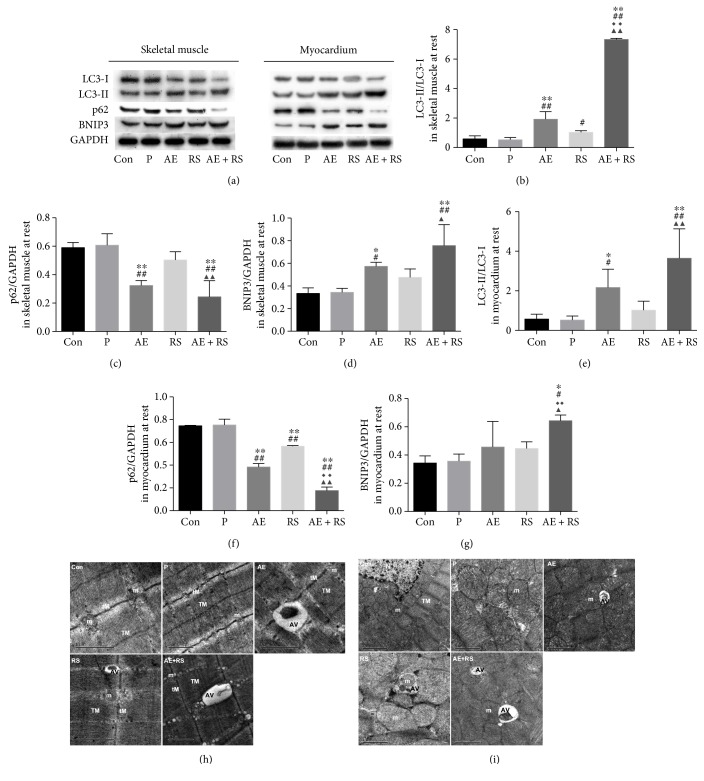
Effects of aerobic exercise and *Rhodiola sacra* supplementation, alone and combined, on the ratio of LC3-II/LC3-I (b), level of p62 (c), and level of BNIP3 (d) in mouse skeletal muscle and the ratio of LC3-II/LC3-I (e), level of p62 (f), and level of BNIP3 (g) in mouse myocardium; bands from the Western blotting analysis (a). Transmission electron micrographs of mouse skeletal (h) and cardiac (i) muscles: TEM image; AV: autophagic vacuole, m: mitochondria, tM: thin myofilament, TM: thick myofilament, *N. nucleus*. Scale bar = 1 or 2 *μ*m. ^∗^*P* < 0.05, ^∗∗^*P* < 0.01 versus Con; ^#^*P* < 0.05, ^##^*P* < 0.01 versus P; ^◆◆^*P* < 0.01 versus AE; ^▲^*P* < 0.05, ^▲▲^*P* < 0.01 versus RS.

To determine whether AE and RS, alone and combined, activate autophagy and mitophagy in mouse SCM, we analyzed the ratio of LC3-II/LC3-I and the expression of p62, which are markers of autophagic activity [33], and the expression of BNIP3, a mitophagy biomarker, by Western blotting [38]. Autophagosomes were detected by TEM. Both AE training and RS supplementation significantly increased the number of autophagosomes, ratio of LC3-II/LC3-I, and level of BNIP3 and decreased the level of p62 compared with that in the Con and P groups (*P* < 0.05). Similarly, the combination of AE and RS further increased the number of autophagosomes, ratio of LC3-II/LC3-I, and level of BNIP3 and decreased the level of p62 in mouse SCM compared with that in the AE or RS groups (*P* < 0.05) (Figure 3).

#### 3.1.4. AE Activates Mitochondrial Dynamics in Skeletal and Cardiac Muscles

**Figure 4:**
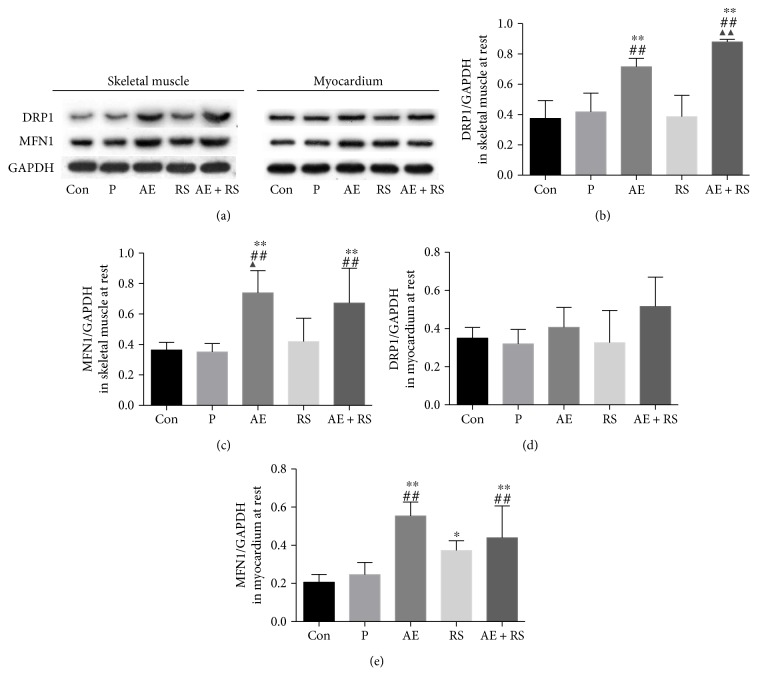
Effects of aerobic exercise and *Rhodiola sacra* supplementation, alone and combined, on the levels of dynamin-related protein 1 (b) and mitochondrial fusion 1 (c) in mouse skeletal muscle and DRP (d) 1 and MFN1 (e) in mouse myocardium; bands from the Western blotting analysis (a). ^∗^*P* < 0.05, ^∗∗^*P* < 0.01 versus Con; ^##^*P* < 0.01 versus P; ^▲^*P* < 0.05, ^▲▲^*P* < 0.01 versus RS.

DRP1 and MFN1 are, respectively, associated with mitochondrial fission and fusion. Therefore, to determine whether AE and RS, alone and combined, stimulate mitochondrial dynamics in SCM, we evaluated the expression levels of these proteins by Western blotting. AE significantly increased the levels of both DRP1 and MFN1 in mouse SCM (*P* < 0.01). However, RS supplementation did not increase the levels of DRP1 and MFN1 in the muscles of mice in the resting state (Figure 4).

#### 3.1.5. The AMPK/PGC-1*α* Signaling Pathway Is a Pivotal Regulator of Mitochondrial Biogenesis

**Figure 5:**
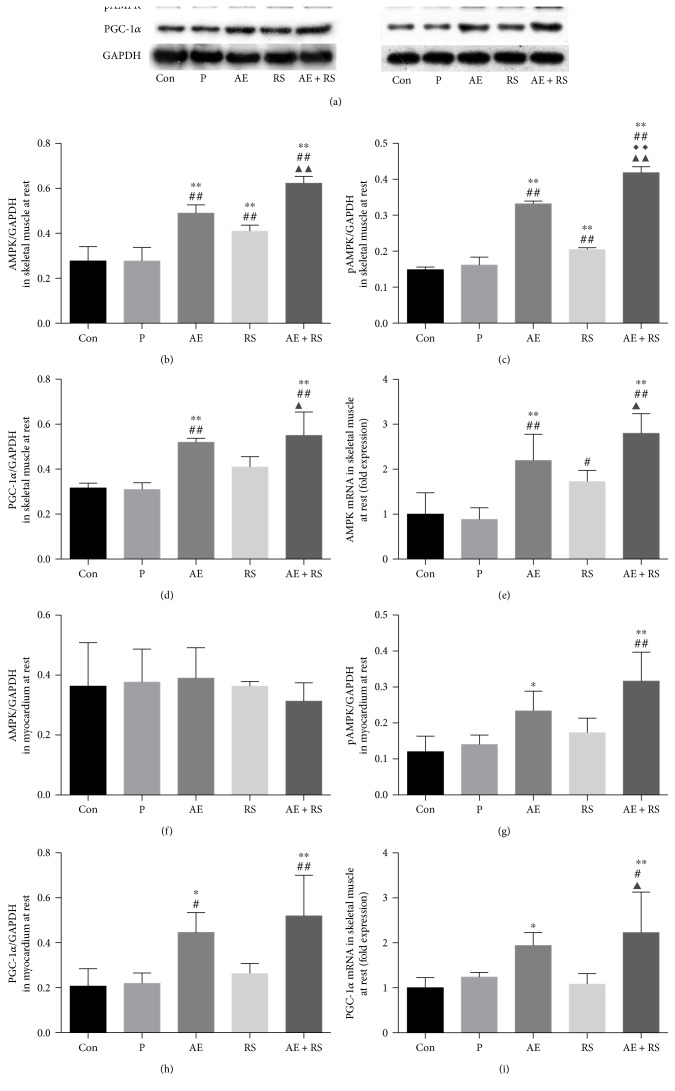
Effects of aerobic exercise, with and without *Rhodiola sacra* supplementation, on the levels of AMPK (b), pAMPK (c), PGC-1*α* (d), AMPK mRNA (e), and PGC-1*α* mRNA (i) in mouse skeletal muscle and AMPK (f), pAMPK (g), and PGC-1*α* (h) in mouse myocardium; bands from the Western blotting analysis (a). ^∗^*P* < 0.05, ^∗∗^*P* < 0.01 versus Con; ^#^*P* < 0.05, ^##^*P* < 0.01 versus P; ^◆◆^*P* < 0.01 versus AE; ^▲^*P* < 0.05, ^▲▲^*P* < 0.01 versus RS.

Therefore, to analyze the effects of AE and RS, alone and combined, on mitochondrial biogenesis in mouse SCM, we analyzed this pathway by Western blotting and RT-qPCR analysis. AE significantly activated the AMPK/PGC-1*α* signaling pathway in SCM, while RS significantly activated this pathway in skeletal muscle. A synergistic effect was detected both in SCM (Figure 5).

### 3.2. Effects of AE and RS, Alone and Combined, on Exhaustive Exercise-Induced Skeletal and Cardiac Muscle Damage and the Underlying Mechanism

#### 3.2.1. AE and RS, Alone and Combined, Ameliorate EE-Induced Skeletal and Cardiac Muscle Damage

**Figure 6:**
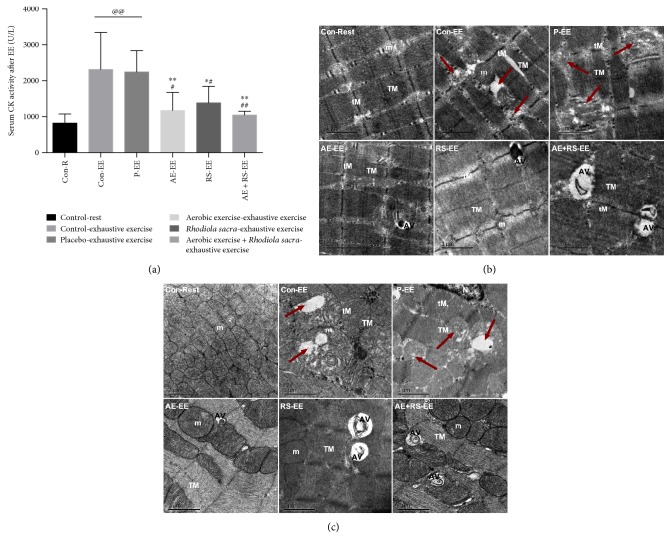
Effects of aerobic exercise and *Rhodiola sacra* supplementation, alone and combined, on skeletal and cardiac muscle damage caused by exhaustive exercise-induced stress; serum creatine kinase activity (a) and transmission electron micrograph of skeletal (b) and cardiac (c) muscles in mice exhausted from exercise; R: rest; EE: exhaustive exercise; Con-Rest: control-rest; Con-EE: control-exhaustive exercise; P-EE: placebo-exhaustive exercise; AE-EE: aerobic exercise-exhaustive exercise; RS-EE: *Rhodiola sacra*-exhaustive exercise; AE + RS-EE: aerobic exercise combined with *Rhodiola sacra*-exhaustive exercise. TEM image shows damaged myofiber or mitochondria (red arrow); AV: autophagic vacuole; m: mitochondria; tM: thin myofilament; TM: thick myofilament; scale bar = 1 or 2 *μ*m; ^@@^*P* < 0.01 versus Con-N, ^∗^*P* < 0.05, ^∗∗^*P* < 0.01 versus Con-EE, ^#^*P* < 0.05, ^##^*P* < 0.01 versus P-EE; *n* = 5 mice.

To demonstrate that EE causes SCM damage and investigate the protective effects of AE and RS, alone and combined, we assessed the ultrastructure of SCM by TEM and determined the activity of serum creatine kinase (CK) using a colorimetric method. SCM from mice subject to EE exhibited characteristics of fiber necrosis, mitochondrion edema, and mitochondrial cristae dissolution and degradation; in addition, serum CK activity in mice exhausted from exercise was 2.8 times higher than that in normal (*n* = 5 mice, *P* < 0.01). Both AE and RS ameliorated SCM damage caused by EE; serum CK activities in mice pretreated with AE or RS were lower than those in the Con and P groups (*P* < 0.05). Moreover, synergistic effects of AE and RS on the amelioration of EE-induced muscle damage were detected. Serum CK activity in mice receiving combined pretreatment with AE and RS supplementation was, respectively, 11% and 24% lower than that in mice pretreated with AE or RS alone (Figure 6).

#### 3.2.2. AE and RS, Alone and Combined, Reduced the Level of Mitochondrial Oxidative Stress in Skeletal and Cardiac Muscles of Mice after EE

**Figure 7:**
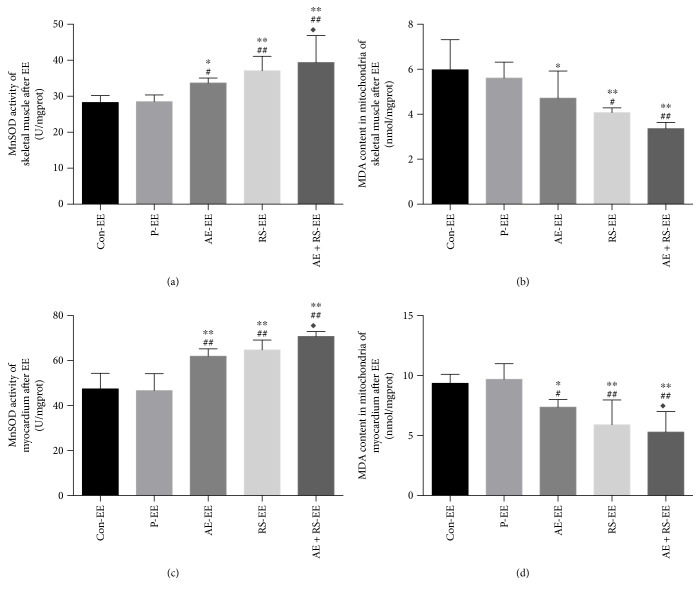
Effects of aerobic exercise and *Rhodiola sacra supplementation*, alone and combined, on MnSOD activity (a) in skeletal muscle and MDA content in mitochondria (b) in skeletal muscle and on MnSOD activity (c) in myocardium and MDA content in mitochondria (c) in myocardium of mice exhausted from exercise. ^∗^*P* < 0.05, ^∗∗^*P* < 0.01 versus Con-EE; ^#^*P* < 0.05, ^##^*P* < 0.01 versus P-EE; ^◆^*P* < 0.05 versus AE-EE.

To determine whether AE and RS supplementation, alone and combined, improved mitochondrial oxidative stress in SCM, we measured manganese superoxide dismutase (MnSOD) activity in SCM and malonaldehyde (MDA) content in mitochondria. Mice pretreated with AE or RS exhibited a 19% to 30% higher MnSOD activity and a 21% to 27% lower MDA content than those in the Con and P groups in skeletal muscle (*P* < 0.05); Mice pretreated with AE or RS exhibited a 31% to 39% higher MnSOD activity and a 21% to 38% lower MDA content than those in the Con and P groups in myocardium (*P* < 0.05). The combination of AE and RS showed a synergistic effect (*P* < 0.05) (Figure 7).

#### 3.2.3. AE and RS, Alone and Combined, Activate Autophagy and Mitophagy in Mouse Skeletal and Cardiac Muscles after EE

**Figure 8:**
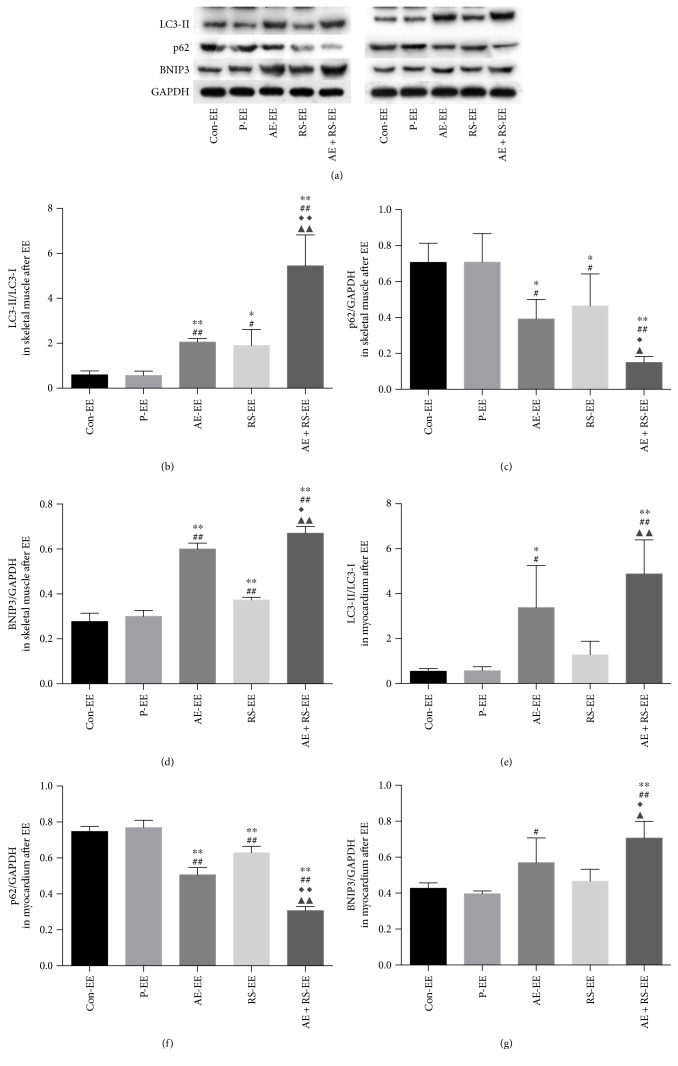
Effects of aerobic exercise and *Rhodiola sacra* supplementation, alone and combined, on the ratio of LC3-II/LC3-I (b), level of p62 (c), and level of BNIP3 (d) in skeletal muscle and on the ratio of LC3-II/LC3-I (e), level of p62 (f), and level of BNIP3 (g) in myocardium of mice exhausted from exercise; bands from the Western blotting analysis (a); ^∗^*P* < 0.05, ^∗∗^*P* < 0.01 versus Con-EE, ^#^*P* < 0.05, ^##^*P* < 0.01 versus P-EE. ^◆^*P* < 0.05, ^◆◆^*P* < 0.01 versus AE-EE. ^▲^*P* < 0.05, ^▲▲^*P* < 0.01 versus RS-EE.

AE significantly increased the number of autophagosomes, ratio of LC3-II/LC3-I, and level of BNIP3 and reduced the level of p62 in SCM of mice exhausted from exercise (*P* < 0.05). RS had the same effect in skeletal muscle (*P* < 0.05). AE and RS were found to have synergistic effects via increasing the ratio of LC3-II/LC3-I and BNIP3 levels and decreasing the p62 level in SCM of mice after EE (*P* < 0.05) (Figures 6(b), 6(c), and 8).

#### 3.2.4. AE and RS, Alone and Combined, Activate Mitochondrial Dynamics in Skeletal Muscle of Mice after Exhaustive Exercise

**Figure 9:**
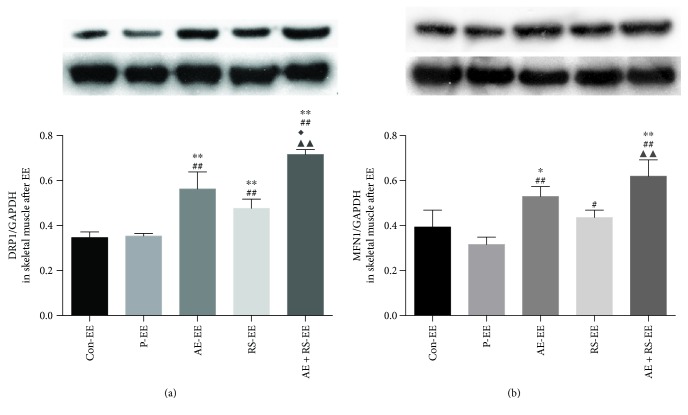
Effects of aerobic exercise and *Rhodiola sacra* supplementation, alone and combined, on the levels of DRP1 (a) and MFN1 (b) in the skeletal muscle of mice exhausted from exercise. ^∗^*P* < 0.05, ^∗∗^*P* < 0.01 versus Con-EE; ^#^*P* < 0.05, ^##^*P* < 0.01 versus P-EE; ^◆^*P* < 0.05 versus AE-EE; ^▲▲^*P* < 0.01 versus RS-EE.

Both AE and RS significantly increased the levels of DRP1 and MFN1 (*P* < 0.05) in skeletal muscle of mice exhausted from exercise (*P* < 0.05); the two factors were found to have a synergistic effect (Figure 9).

#### 3.2.5. AE and RS, Alone and Combined, Activate Mitochondrial Biogenesis in Skeletal Muscle of Mice after Exhaustive Exercise

**Figure 10:**
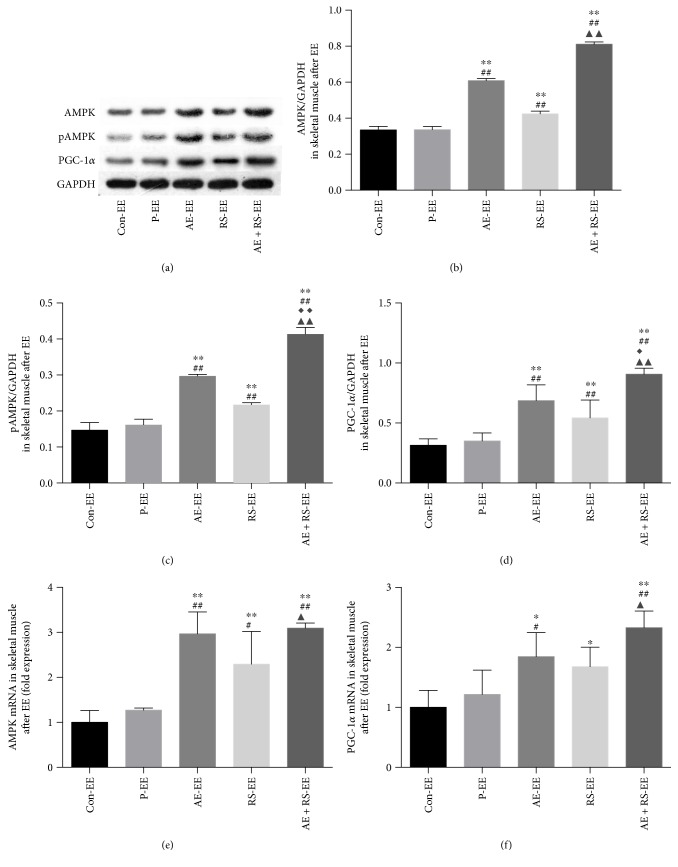
Effects of aerobic exercise and *Rhodiola sacra* supplementation, alone and combined, on the levels of AMPK (b), pAMPK (c), PGC-1*α* (d), AMPK mRNA (e), and PGC-1*α* mRNA (f) in skeletal muscle of mice exhausted from exercise; bands from the Western blotting analysis (a). ^∗^*P* < 0.05, ^∗∗^*P* < 0.01 versus Con-EE; ^#^*P* < 0.05, ^##^*P* < 0.01 versus P-EE; ^◆^*P* < 0.05, ^◆◆^*P* < 0.01 versus AE-EE; ^▲^*P* < 0.05, ^▲▲^*P* < 0.01 versus RS-EE.

Both AE and RS activated the AMPK/PGC-1*α* signaling pathway in skeletal muscle of mice exhausted from exercise (*P* < 0.05); a synergistic effect was detected (*P* < 0.05) (Figure 10).

## 4. Discussion

In this study, we showed that AE and RS supplementation increased EC in mice and ameliorated SCM damage induced by EE, and a synergistic effect was detected. The corresponding mechanism is involved in the enhancement of MQC in mouse SCM, including the activation of mitophagy, mitochondrial dynamics, and biogenesis.

### 4.1. AE and RS, Alone and Combined, Improved Exercise Capacity in Mice through the Enhancement of Mitochondrial Quality Control in SCM

In the first part of this study, we investigated the effects of AE and RS, alone and combined, on EC in mice and the underlying mechanism with a focus on MQC. EC is a potentially stronger predictor of mortality than established risk factors such as smoking, hypertension, high cholesterol, and type 2 diabetes mellitus [3, 4], and it has been recommended for use as a clinical vital sign by the American Heart Association [5]. We found that both AE and RS significantly increased the duration of forced weight-loaded swimming of mice, which is a measure of EC [35]. These results are in accordance with those of previous studies [39, 40]. Moreover, we report, for the first time, that AE combined with RS supplementation synergistically induce a significant increase in EC in mice.

Mitochondria play a vital role in enhancing EC through the regulation of substrate metabolism and energy production, as well as their influence on skeletal muscle size and function [41]. On the organellar level, MQC is a network of interactions between mitophagy, mitochondrial dynamics, and biogenesis [15]. This dynamic process allows the mitochondria to share components, such as mitochondrial DNA, and to eliminate damaged components through mitophagy. Mitochondrial biogenesis and mitophagy contribute to the homeostasis of mitochondria within cells [41]. Any defect in mitophagy, mitochondrial dynamics, or biogenesis will lead to mitochondrial dysfunction, resulting in low EC [42]. In this study, we found that AE significantly increases the expression of CS and activates mitophagy, mitochondrial dynamics, and biogenesis in skeletal muscle. Further, AE was found to induce myocardial autophagy and mitochondrial biogenesis in the myocardium. These results indicate that AE enhances the function of mitochondria and MQC of SCM.

RS is a member of the *Rhodiola* family that is grown in Tibet in China [27]; this natural plant pharmaceutical is well known for its physiological benefits such as elimination of fatigue [43], enhancement of EC [28], and prolongation of lifespan [29, 30]. Several recent studies have demonstrated that RS activates autophagy [44] and mitochondrial biogenesis in endothelial cells [33] and the myocardium in rats [45]. Here, we report for the first time that RS enhances the function of mitochondria in SCM and activates autophagy and mitochondrial biogenesis in mouse skeletal muscle. Notably, RS was found to be as effective as AE in improving EC; however, RS alone was not as effective as AE in activating autophagy. This is likely because the mechanism by which RS improves EC is not limited to activation of autophagy; for example, RS also increases hepatic glycogen synthesis [46]. In contrast to previously reported findings [45], the present study indicated that RS did not significantly increase mitochondrial biogenesis in myocardium. This difference may be attributed to the animal model used: it is possible that the effects of RS on mitochondrial biogenesis are more obvious in damaged myocardium [45] than in normal myocardium. This hypothesis is in accordance with that of a previous study [27] in which *Rhodiola* was categorized as an adaptogen owing to its ability to increase resistance to a variety of chemical, biological, and physical stressors. Our results also provide additional evidence for the adaptogen characteristic of RS in strengthening mitochondrial biogenesis in myocardium in mice subject to AE, which is a type of physical stressor. However, the mechanism underlying the adaptogen characteristic of RS requires further investigation. These results suggest that RS enhances the function of mitochondria and the MQC in SCM, especially following stress.

In addition, for the first time, we showed that AE and RS have a synergistic effect on improving EC and mitochondrial function in SCM. Mice treated with a combination of AE and RS exhibited the longest duration of forced weight-loaded swimming; this was 13.7 times that of the AE group and 1.3 times that of the RS group. Furthermore, the former exhibited greater muscle strength than the AE- or RS-alone groups. Our results indicate that at least two vital factors contribute to this synergistic effect: first, the combination of AE and RS further activates mitophagy in skeletal muscle, which promotes the elimination of dysfunctional or unnecessary mitochondria and supports skeletal muscle plasticity in response to AE. Second, the combination further activates autophagy and mitochondrial biogenesis in the myocardium, which contributes to improving myocardial energy metabolism. These findings suggest that AE and RS have a synergistic effect on improving MQC in SCM. However, the mechanism by which the synergistic effect of AE and RS increases EC is not limited to the improvement of MQC in SCM, as the increase in EC was significantly higher than that during mitophagy, mitochondrial dynamics, and biogenesis in SCM. This study focused on the MQC in the SCM; our next study will aim to analyze the mechanisms by which the synergistic effect of AE and RS increases EC.

### 4.2. AE and RS, Alone and Combined, Ameliorate Exhaustive Exercise-Induced Skeletal and Cardiac Muscle Damage through the Enhancement of Mitochondrial Quality Control

In the second part of this study, we investigated the protective effect of AE and RS, alone and combined, on EE-induced SCM damage and examined the corresponding mechanism, with a focus on MQC. We found that the level of serum CK, an indicator of muscle damage, significantly increased in mice exhausted by exercise. Morphological analysis using TEM showed that mice exhausted by exercise exhibited fiber necrosis, mitochondrion edema, and mitochondrial cristae dissolution and degradation in SCM. These results indicate that EE, a fatal stressor, caused SCM damage; this finding is in accordance with those of previous studies [45, 47].

EE-induced SCM damage is associated with oxidative stress, which leads to damage to the mitochondrial membrane [48]. Damaged mitochondrial membranes promote the opening of the permeability transition pores, resulting in mitochondrial swelling and the release of apoptosis-inducing factor into the cytosol. Once released, these factors initiate apoptotic signaling and contribute to muscle damage [20]. Mitophagy combined with mitochondrial fission, a process of mitochondrial dynamics, promotes the isolation and elimination of damaged components of mitochondria [15], which is a vital process for the maintenance of cell homeostasis. In addition, mitochondria are produced by mitochondrial biogenesis, which is regulated by PGC-1*α*. These organelles undergo cycles of fusion, mediated by mitofusin (MFN) 1, to form elongated mitochondrial networks [20, 49]. The activation of these three processes and their balance are vital in protecting against EE-induced muscle damage caused by mitochondrial oxidative stress. The uncoupling of mitophagy and mitochondrial biogenesis during ageing contributes to the overproliferation of damaged mitochondria and decrease in cellular function [50].

Recent studies have suggested that mitochondria are a target for exercise-induced cardioprotection [51]. Similarly, Vainshtein et al. proposed that exercise increases mitochondrial turnover and that this is partly coordinated by PGC-1*α* [42]. In order to address the question of whether AE ameliorates EE-induced muscle damage, reduces the level of oxidative stress in SCM, and enhances myocardial protection in mice exhausted from exercise, we analyzed the ultrastructure of SCM and measured CK levels and MnSOD activity in SCM and the MDA content in the mitochondria of SCM of mice exhausted from exercise. We found that AE significantly ameliorated SCM damage induced by EE, improved muscle mitochondrial oxidative stress, and enhanced myocardial protection in these mice. Furthermore, AE significantly activated autophagy and mitophagy in SCM and promoted mitochondrial dynamics and biogenesis in the skeletal muscle of mice exhausted from exercise. These results suggest that the enhancement of MQC is involved in the protective effects of AE on EE-induced SCM damage caused by mitochondrial oxidative stress.

RS is a medicinal plant with demonstrated adaptogenic properties [27]. Several recent studies showed that RS root extract improved stress tolerance in silkworm (*Bombyx mori*) [30], protected C2C12 myotubes against peroxide-induced oxidative stress through the modulation of the molecular chaperone HSP70 [52], and maintained cell membrane permeability by resisting oxidative stress caused by cold, hypoxia, and restraint [53]. Moreover, Abidov et al. concluded that *Rhodiola* enhances mitochondrial function [54]. Liu et al. proposed that *Rhodiola* induces autophagy [32]. In this study, we showed that RS significantly ameliorated SCM damage and improved SCM mitochondrial oxidative stress both induced by EE. Moreover, we report for the first time that RS significantly activated autophagy and mitophagy in SCM and promoted mitochondrial dynamics and biogenesis in skeletal muscle of mice exhausted from exercise. RS was shown to be as effective as AE in ameliorating EE-induced muscle damage; however, RS alone was not as effective as AE in activating autophagy. This is likely because the mechanism by which RS reduces EE-induced muscle damage is not limited to the activation of autophagy, but also include other mechanisms; for example, RS increases the expression of muscle-protective factors [52]. These results indicate that the enhancement of MQC is involved in the protective effects of RS on EE-induced SCM damage caused by mitochondrial oxidative stress.

In addition, for the first time, we showed that AE combined with RS had synergistic protective effects on ameliorating EE-induced SCM damage. Mice treated with the combination of AE and RS exhibited 11% and 24% lower activity levels of serum CK and less damage than those treated with AE or RS alone. Our data indicate that at least two vital factors contribute to this synergistic protective effect. First, AE combined with RS activates autophagy and mitophagy, mitochondrial dynamics, and biogenesis in skeletal muscle. Second, the combination further activates autophagy and mitophagy in myocardium. These findings suggest that the synergistic effect of AE and RS in ameliorating SCM damage caused by EE is related to the enhancement of MQC. However, the mechanism involved is not limited to improve MQC in SCM. This study focused on MQC in SCM; analyses of the mechanism of the synergistic protective effect of AE and RS will be performed in our next study.

It should be noted that this study did not use an inhibitor or agonist to verify the results; we aim to overcome this limitation in our next study.

## 5. Conclusion

**Figure 11:**
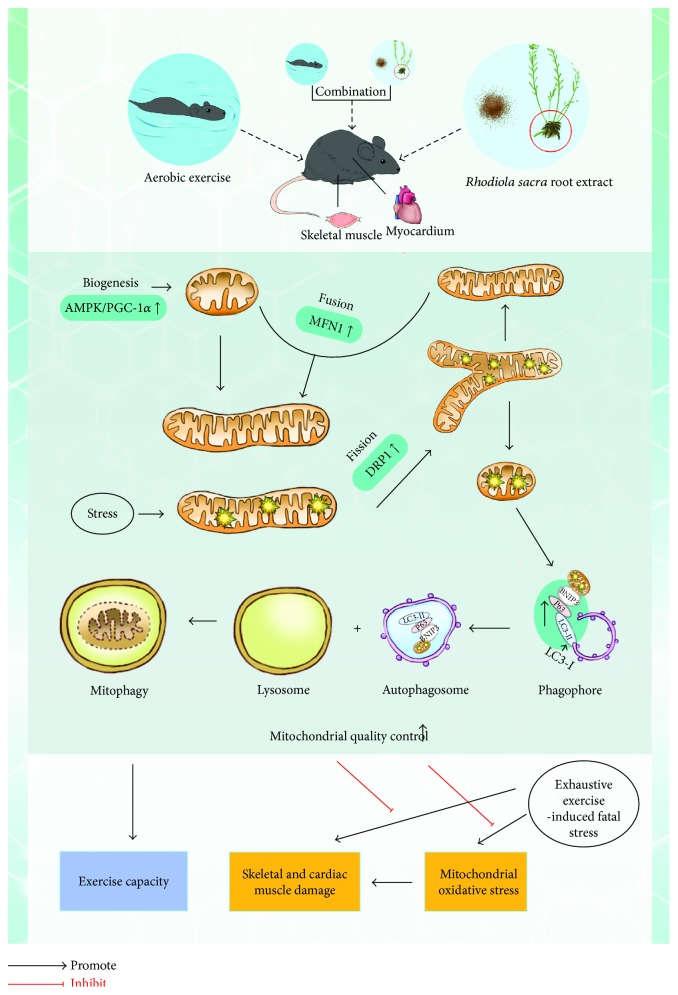
Schematic diagram of the putative mechanism by which aerobic exercise and *Rhodiola sacra*, alone and combined, improve exercise capacity and ameliorate muscle damage induced by exhaustive exercise in mice: Aerobic exercise and *Rhodiola sacra*, alone and combined, enhanced mitochondrial quality control, including mitophagy, mitochondrial dynamics, and biogenesis, in SCM of mice. These effects contribute to the enhancement of mitochondrial function and exercise capacity and protection against SCM damage caused by excessive mitochondrial oxidative stress resulting from EE.

In this study, we showed that AE and RS significantly increase EC in mice and ameliorate EE-induced SCM damage caused by mitochondrial oxidative stress; further, it was demonstrated, for the first time, that RS supplementation and AE exert synergistic effects. Moreover, the present study is the first to show that AE and RS enhance MQC, both in the resting state and after EE, through the activation of mitophagy, mitochondrial dynamics, and biogenesis in skeletal or cardiac muscles (Figure 11). AE combined with RS supplementation thus provides a novel strategy to reduce the risks of all-cause mortality and adverse cardiovascular events in patients with CVD.

## Figures and Tables

**Table 1:** Sequence of primers used for RT-qPCR assays.

Genes	Primer sequences	
	Forward primer (5′→3′)	Reverse primer (5′→3′)
*AMPK*	CGGGGTCATTCTCTATGCTT	TTTAAACCACTCGTGTTCCCT
*PGC-1α*	ATGCCTGCCCAGTACCTGA	CGCAGCTCACAAAATACTGTCC
*GAPDH*	GCGACTTCAACAGCAACTCCC	CACCCTGTTGCTGTAGCCGTA

## References

[1] Lozano R., Naghavi M., Foreman K. (2012). Global and regional mortality from 235 causes of death for 20 age groups in 1990 and 2010: a systematic analysis for the Global Burden of Disease Study 2010. *The Lancet*.

[2] Korpelainen R., Lämsä J., Kaikkonen K. M. (2016). Exercise capacity and mortality – a follow-up study of 3033 subjects referred to clinical exercise testing. *Annals of Medicine*.

[3] Swift D. L., Lavie C. J., Johannsen N. M. (2013). Physical activity, cardiorespiratory fitness, and exercise training in primary and secondary coronary prevention. *Circulation Journal*.

[4] Myers J., McAuley P., Lavie C. J., Despres J. P., Arena R., Kokkinos P. (2015). Physical activity and cardiorespiratory fitness as major markers of cardiovascular risk: their independent and interwoven importance to health status. *Progress in Cardiovascular Diseases*.

[5] Ross R., Blair S. N., Arena R. (2016). Importance of assessing cardiorespiratory fitness in clinical practice: a case for fitness as a clinical vital sign: a scientific statement from the American Heart Association. *Circulation*.

[6] Du J., Zhang D., Yin Y. (2016). The personality and psychological stress predict major adverse cardiovascular events in patients with coronary heart disease after percutaneous coronary intervention for five years. *Medicine*.

[7] Dominguez-Rodriguez A., Rodríguez S., Abreu-Gonzalez P., Avanzas P., Juarez-Prera R. A. (2015). Black carbon exposure, oxidative stress markers and major adverse cardiovascular events in patients with acute coronary syndromes. *International Journal of Cardiology*.

[8] Vassalle C., Bianchi S., Battaglia D., Landi P., Bianchi F., Carpeggiani C. (2012). Elevated levels of oxidative stress as a prognostic predictor of major adverse cardiovascular events in patients with coronary artery disease. *Journal of Atherosclerosis and Thrombosis*.

[9] Addison P. D., Neligan P. C., Ashrafpour H. (2003). Noninvasive remote ischemic preconditioning for global protection of skeletal muscle against infarction. *American Journal of Physiology - Heart and Circulatory Physiology*.

[10] Schmidt M. R., Smerup M., Konstantinov I. E. (2007). Intermittent peripheral tissue ischemia during coronary ischemia reduces myocardial infarction through a K_ATP_-dependent mechanism: first demonstration of remote ischemic perconditioning. *American Journal of Physiology - Heart and Circulatory Physiology*.

[11] Malhotra R., Dhakal B. P., Eisman A. S. (2016). Pulmonary vascular distensibility predicts pulmonary hypertension severity, exercise capacity, and survival in heart failure. *Circulation: Heart Failure*.

[12] Blomster J. I., Svedlund S., Westergren H. U., Gan L. M. (2016). Coronary flow reserve as a link between exercise capacity, cardiac systolic and diastolic function. *International Journal of Cardiology*.

[13] Van Remoortel H., De Buck E., Compernolle V., Deldicque L., Vandekerckhove P. (2017). The effect of a standard whole blood donation on oxygen uptake and exercise capacity: a systematic review and meta-analysis. *Transfusion*.

[14] Wu Y. T., Wu S. B., Wei Y. H. (2014). Roles of sirtuins in the regulation of antioxidant defense and bioenergetic function of mitochondria under oxidative stress. *Free Radical Research*.

[15] Figge M. T., Osiewacz H. D., Reichert A. S. (2013). Quality control of mitochondria during aging: is there a good and a bad side of mitochondrial dynamics?. *BioEssays*.

[16] Cho D. H., Nakamura T., Fang J. (2009). S-nitrosylation of Drp1 mediates *β*-amyloid-related mitochondrial fission and neuronal injury. *Science*.

[17] Zhu Y., Massen S., Terenzio M. (2013). Modulation of serines 17 and 24 in the LC3-interacting region of Bnip3 determines pro-survival mitophagy versus apoptosis. *Journal of Biological Chemistry*.

[18] Pankiv S., Clausen T. H., Lamark T. (2007). p62/SQSTM1 binds directly to Atg8/LC3 to facilitate degradation of ubiquitinated protein aggregates by autophagy. *Journal of Biological Chemistry*.

[19] Jäger S., Handschin C., St-Pierre J., Spiegelman B. M. (2007). AMP-activated protein kinase (AMPK) action in skeletal muscle via direct phosphorylation of PGC-1*α*. *Proceedings of the National Academy of Sciences of the United States of America*.

[20] Carter H. N., Chen C. C., Hood D. A. (2015). Mitochondria, muscle health, and exercise with advancing age. *Physiology*.

[21] Koltai E., Hart N., Taylor A. W. (2012). Age-associated declines in mitochondrial biogenesis and protein quality control factors are minimized by exercise training. *American Journal of Physiology - Regulatory, Integrative and Comparative Physiology*.

[22] Ferraro E., Giammarioli A. M., Chiandotto S., Spoletini I., Rosano G. (2014). Exercise-induced skeletal muscle remodeling and metabolic adaptation: redox signaling and role of autophagy. *Antioxidants & Redox Signaling*.

[23] Taylor C. W., Ingham S. A., Hunt J. E., Martin N. R., Pringle J. S., Ferguson R. A. (2016). Exercise duration-matched interval and continuous sprint cycling induce similar increases in AMPK phosphorylation, PGC-1*α* and VEGF mRNA expression in trained individuals. *European Journal of Applied Physiology*.

[24] Li L., Xiao L., Hou Y. (2016). Sestrin2 silencing exacerbates cerebral ischemia/reperfusion injury by decreasing mitochondrial biogenesis through the AMPK/PGC-1*α* pathway in rats. *Scientific Reports*.

[25] Marongiu E., Crisafulli A. (2014). Cardioprotection acquired through exercise: the role of ischemic preconditioning. *Current Cardiology Reviews*.

[26] Smuder A. J., Kavazis A. N., Min K., Powers S. K. (2011). Exercise protects against doxorubicin-induced oxidative stress and proteolysis in skeletal muscle. *Journal of Applied Physiology*.

[27] Kelly G. S. (2001). *Rhodiola rosea*: a possible plant adaptogen. *Alternative Medicine Review*.

[28] Parisi A., Tranchita E., Duranti G. (2010). Effects of chronic *Rhodiola rosea* supplementation on sport performance and antioxidant capacity in trained male: preliminary results. *Journal of Sports Medicine and Physical Fitness*.

[29] Jafari M., Felgner J. S., Bussel I. I. (2007). *Rhodiola*: a promising anti-aging Chinese herb. *Rejuvenation Research*.

[30] Chen C., Song J., Chen M. (2016). *Rhodiola rosea* extends lifespan and improves stress tolerance in silkworm, *Bombyx mori*. *Biogerontology*.

[31] Huang S. C., Lee F. T., Kuo T. Y., Yang J. H., Chien C. T. (2009). Attenuation of long-term *Rhodiola rosea* supplementation on exhaustive swimming-evoked oxidative stress in the rat. *The Chinese Journal of Physiology*.

[32] Liu Z., Li X., Simoneau A. R., Jafari M., Zi X. (2012). *Rhodiola rosea* extracts and salidroside decrease the growth of bladder cancer cell lines via inhibition of the mTOR pathway and induction of autophagy. *Molecular Carcinogenesis*.

[33] Xing S., Yang X., Li W. (2014). Salidroside stimulates mitochondrial biogenesis and protects against H_2_O_2_-induced endothelial dysfunction. *Oxidative Medicine and Cellular Longevity*.

[34] Matsumoto K., Ishihara K., Tanaka K., Inoue K., Fushiki T. (1996). An adjustable-current swimming pool for the evaluation of endurance capacity of mice. *Journal of Applied Physiology*.

[35] Yeh T. S., Chuang H. L., Huang W. C., Chen Y. M., Huang C. C., Hsu M. C. (2014). *Astragalus membranaceus* improves exercise performance and ameliorates exercise-induced fatigue in trained mice. *Molecules*.

[36] Cheng T. L., Liao C. C., Tsai W. H. (2009). Identification and characterization of the mitochondrial targeting sequence and mechanism in human citrate synthase. *Journal of Cellular Biochemistry*.

[37] Moat S. J., Korpimäki T., Furu P. (2017). Characterization of a blood spot creatine kinase skeletal muscle isoform immunoassay for high-throughput newborn screening of Duchenne muscular dystrophy. *Clinical Chemistry*.

[38] Zhang T., Xue L., Li L. (2016). BNIP3 protein suppresses PINK1 kinase proteolytic cleavage to promote mitophagy. *Journal of Biological Chemistry*.

[39] Sobol N. A., Hoffmann K., Frederiksen K. S. (2016). Effect of aerobic exercise on physical performance in patients with Alzheimer’s disease. *Alzheimer’s & Dementia*.

[40] Noreen E. E., Buckley J. G., Lewis S. L., Brandauer J., Stuempfle K. J. (2013). The effects of an acute dose of *Rhodiola rosea* on endurance exercise performance. *Journal of Strength and Conditioning Research*.

[41] Russell A. P., Foletta V. C., Snow R. J., Wadley G. D. (2014). Skeletal muscle mitochondria: a major player in exercise, health and disease. *Biochimica et Biophysica Acta (BBA) - General Subjects*.

[42] Vainshtein A., Tryon L. D., Pauly M., Hood D. A. (2015). Role of PGC-1*α* during acute exercise-induced autophagy and mitophagy in skeletal muscle. *American Journal of Physiology - Cell Physiology*.

[43] Petkov V. D., Yonkov D., Mosharoff A. (1986). Effects of alcohol aqueous extract from *Rhodiola rosea L.* roots on learning and memory. *Acta Physiologica et Pharmacologica Bulgarica*.

[44] Zheng X. T., Wu Z. H., Wei Y. (2017). Induction of autophagy by salidroside through the AMPK-mTOR pathway protects vascular endothelial cells from oxidative stress-induced apoptosis. *Molecular and Cellular Biochemistry*.

[45] Ping Z., Zhang L. F., Cui Y. J. (2015). The protective effects of salidroside from exhaustive exercise-induced heart injury by enhancing the *PGC-1*α–*NRF1*/*NRF2* pathway and mitochondrial respiratory function in rats. *Oxidative Medicine and Cellular Longevity*.

[46] Lin K. T., Hsu S. W., Lai F. Y., Chang T. C., Shi L. S., Lee S. Y. (2016). *Rhodiola crenulata* extract regulates hepatic glycogen and lipid metabolism via activation of the AMPK pathway. *BMC Complementary and Alternative Medicine*.

[47] Li H., Miao W., Ma J. (2016). Acute exercise-induced mitochondrial stress triggers an inflammatory response in the myocardium via NLRP3 inflammasome activation with mitophagy. *Oxidative Medicine and Cellular Longevity*.

[48] Popovic L. M., Mitic N. R., Radic I. (2012). The effect of exhaustive exercise on oxidative stress generation and antioxidant defense in guinea pigs. *Advances in Clinical and Experimental Medicine*.

[49] Kluge M. A., Fetterman J. L., Vita J. A. (2013). Mitochondria and endothelial function. *Circulation Research*.

[50] Palikaras K., Lionaki E., Tavernarakis N. (2015). Coordination of mitophagy and mitochondrial biogenesis during ageing in *C. elegans*. *Nature*.

[51] Ascensão A., Lumini-Oliveira J., Oliveira P. J., Magalhães J. (2011). Mitochondria as a target for exercise-induced cardioprotection. *Current Drug Targets*.

[52] Hernández-Santana A., Pérez-López V., Zubeldia J. M., Jiménez-del-Rio M. (2014). A *Rhodiola rosea* root extract protects skeletal muscle cells against chemically induced oxidative stress by modulating heat shock protein 70 (HSP70) expression. *Phytotherapy Research*.

[53] Gupta V., Lahiri S. S., Sultana S., Tulsawani R. K., Kumar R. (2010). Anti-oxidative effect of *Rhodiola imbricata* root extract in rats during cold, hypoxia and restraint (C–H–R) exposure and post-stress recovery. *Food and Chemical Toxicology*.

[54] Abidov M., Crendal F., Grachev S., Seifulla R., Ziegenfuss T. (2003). Effect of extracts from *Rhodiola rosea* and *Rhodiola crenulata (Crassulaceae)* roots on ATP content in mitochondria of skeletal muscles. *Bulletin of Experimental Biology and Medicine*.

